# Supplementary figures and images for: DLEU2 facilitates bladder cancer progression through miR-103a-2-5p/SOS1 axis
Source: PeerJ. 2025 Apr 8;13:e18995. doi: 10.7717/peerj.18995 (PMC11988102; doi:10.7717/peerj.18995)

Figure 6E

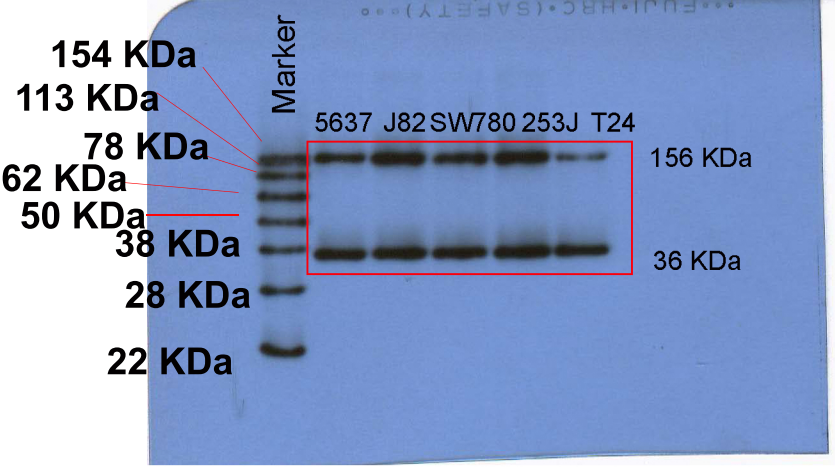

6F

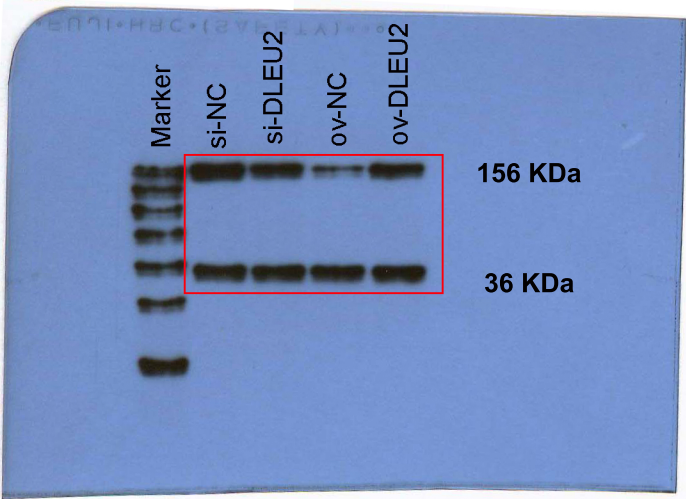

6G

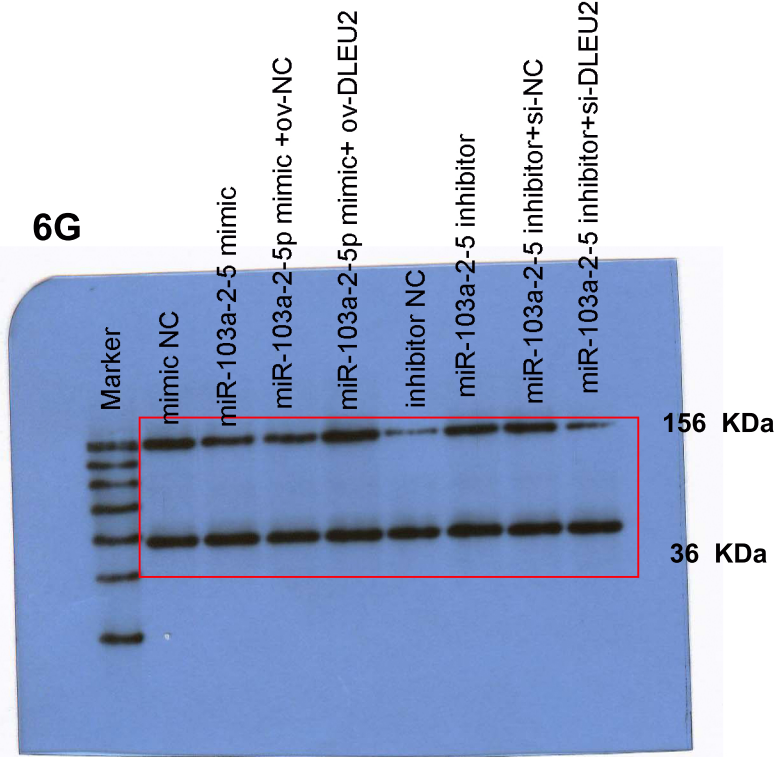

Figure 7A

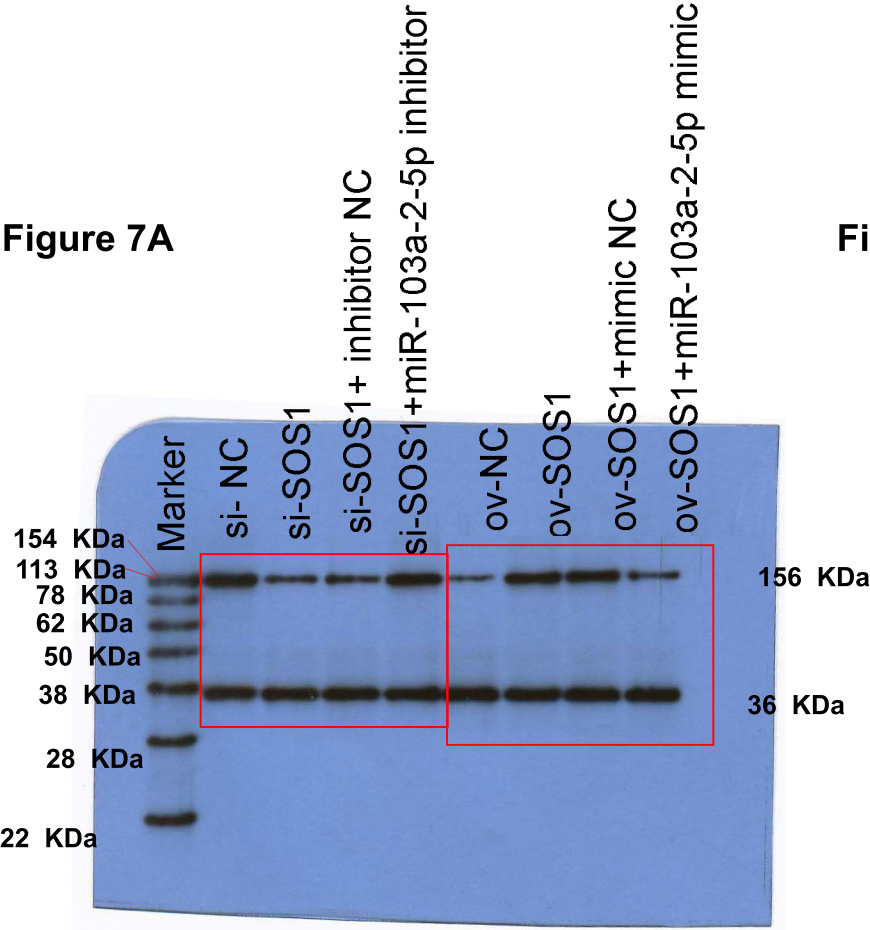

Figure 8A

Supplement: Supplemental Information 1 [file peerj-13-18995-s001.pdf]
